# Anti-colorectal cancer activity of constructed oleogels based on encapsulated bioactive canola extract in lecithin for edible semisolid applications

**DOI:** 10.1038/s41598-025-88488-1

**Published:** 2025-02-10

**Authors:** Engy M. Akl, Ahmed A. Abd-Rabou, Ayat F. Hashim

**Affiliations:** 1 Fats and Oils Department, Food Industries and Nutrition Research Institute, National Research Centre, 33 El Bohouth St., Dokki, P.O.12622, Giza, Egypt; 2 Hormones Department, Medical Research and Clinical Studies Institute, National Research Centre, Cairo, Egypt

**Keywords:** Anti-colorectal cancer, Oleogels, Bioactive extract, Pumpkin seed oil, Antioxidant activity, Biochemistry, Biotechnology, Cancer, Chemistry

## Abstract

Globally, colorectal cancer ranks second in women and third in men. Hydrophilic anticancer agents have limited use in lipid systems due to their weak solubility. Therefore, this study aimed to develop oleogels based on pumpkin seed oil (R1) and hydrophilic bioactive canola extract (BCE or R2) that were extracted from canola meal by-products. BCE was effectively dispersed in oleogels through the encapsulation of BCE with various concentrations (0.08, 0.2, and 0.4%) in soy lecithin to form BCE gelling agents. Four formulations (F1 as plain, F2-F4 with different concentrations of BCE) were produced using two gelators (BCE gelling agent and beeswax). The oxidative stability, microstructure, FTIR, antioxidant activity, and time-dependent experiment were investigated. The cytotoxicity against colorectal HCT116 and Caco-2 cancer cell lines in vitro was evaluated. The anti-apoptotic PI3k and COX-2 protein expressions were also assessed. The peroxide, p-anisidine, and total oxidation values of F4 were 7.85, 26.66, and 42.35, respectively, during 60 days at 60 ± 2 °C. The antioxidant activity values of F4 were 74.40% for DPPH, 54.28% for ABTS, and 5.77 mg/g for FRAP. F4 demonstrated the highest significant cytotoxic effects on cancerous cells, particularly in the Caco-2 cells with 1.40- and 1.41-fold increases compared to R2 and the positive control doxorubicin, respectively. PI3k and COX-2 expression levels were down-regulated while iNOS activity was up-regulated in both cells, with very high down-regulation recorded for F4 in Caco-2 cells. This study developed a method for producing stable lipid products loaded with hydrophilic antioxidants that may be used as an anti-colorectal platform.

**Supplementary Information:**

The online version contains supplementary material available at 10.1038/s41598-025-88488-1.

## Introduction

Colorectal cancer is the second most frequent cancer in women and the third most common cancer in men globally in terms of incidence^1^. Elevated antioxidant polyphenol concentrations can lower the quantity of lipid peroxidation products that may cause cancer when stored^2,3^. They may also positively alter epigenetic modifications and the expression pattern of miRNA, which could lower the risk of developing colorectal cancer^4,5^. A sharp growth in production over the last ten years has made canola seeds (Brassica napus) one of the most significant oilseed crops in the world. Canola has a significantly higher phenolic content than other oilseeds; it is made up of both free and esterified phenolic acids, which are mostly kept in the canola meal after the oil is, extracted. Thousands of metric tons of defatted canola meal by-products are disposed of annually as part of the oil waste sector, even though it may be a substantial source of phenolic and antioxidant compounds^6,7^.

Phytochemical-rich extracts enable the development of natural products with enhanced stability and high levels of antioxidants that are good for humans^8,9^. The existence of unsaturated bonds in the chemical structure of phenolic compounds renders them susceptible to oxidizing factors including light, heat, oxygen, and moisture, which is a crucial consideration when using them. In actuality, exposure to the aforementioned circumstances may result in structural alterations^10^. One potential substitute for preserving the stability of polyphenols throughout food processing appears to be microencapsulation. Lyophilization is the most effective method for encapsulating phytochemicals due to its low processing temperature^11^. Soybean lecithin is a naturally occurring amphiphilic substance made up of many phospholipid derivatives. According to^12^, lecithin possesses both lipophilic and hydrophilic characteristics. While the hydrophilic heads usually interact with water to promote dispersion, the hydrophobic section can adhere to the surface of oil droplets. Lecithin demonstrated a positive synergistic relationship with phenolics and tocopherols^13^. The use of soy lecithin as a coating material has been studied for nanoencapsulating pomegranate peel ellagitannin extracts^14^.

Pumpkin seed oil has the potential to support a healthy human diet due to its high content of monounsaturated and polyunsaturated fatty acids^15^. Moreover, it exhibits immunomodulatory, antimicrobial, antihypertensive, anticancer, and antihypercholesterolemic properties^16^. Pumpkin seed oil can oxidize easily due to the high oxidative degradation susceptibility of polyunsaturated fatty acids^17^. Partially hydrogenated oils (PHOs), often known as trans-fats, were declared ineligible for the GRAS classification by the U.S. Food and Drug Administration (FDA) in 2015.PHOs have a history of usage in the food industry since they are semisolid fats. However, it has been found that trans-fats pose a serious risk to health because they have been linked to the development of cardiovascular disease^18,19^. As a result, it is crucial to reduce lipid oxidation since it can cause food quality to decline. In addition, there is growing interest in finding healthy alternative methods for oil structure.

One such technique is organogelation, which is the formation of a three-dimensional network to immobilize an organic liquid. It has been shown that organogelation, such as the use of carnauba wax and monoglyceride in pomegranate seed oil, beeswax and monoglyceride in hazelnut oil, sunflower wax, and beeswax in olive oil, can postpone edible oil oxidation^20^. Marangoni and Garti (2011) produced food-grade organogelators such as lecithin, phytosterols, and oryzanol^21^. The use of edible lecithin-based oleogels in the food sector is constrained in several ways. Lecithin oleogels are often categorized as weak organogels by^22^. Wax-based oleogels are highly effective since they can develop in a well-formed network with potent oil-binding qualities even at low concentrations^23^. Consequently, the development of a more stable phospholipid-based oleogel would be advantageous to the food sector as a defense system against oxidation and as a healthy oil structure. In the current work, we extracted bioactive compounds from canola meal waste and microencapsulated them in varying amounts in soy lecithin matrix to produce BCE gelling agents. Afterward, oleogels were prepared using two gelators, BCE gelling agents, and beeswax. The microstructure, FTIR, oxidative stability, antioxidant activity (DPPH, ABTS, and FRAP), and time-dependent experiment were all assessed. Finally, the produced oleogels were studied against colorectal cancer in vitro.

## Materials and methods

### Materials

2,2′-azinobis-(3-ethylbenzothiazoline-6-sulfonic acid, ABTS) was supplied from Bio Basic (Canada). Potassium hexacyanoferrate, gallic acid, trichloroacetic acid (TCA),2,2-Diphenyl-1-picrylhydrazyl (DPPH, 90%), and Folin-ciocalteu reagent were purchased from Sigma (St. Louis, MO, USA). Butylated hydroxytoluene (BHT) was obtained from Nice Chemicals Pvt. Ltd (India). Sodium carbonate, sodium bicarbonate, and Potassium persulfate were purchased from Adwic Company, Egypt. Soy lecithin was bought from Carl Roth (Karlsruhe, Germany). Pumpkin seed oil was purchased from the oil extraction unit, National Research Centre, Egypt. Beeswax (BW) was obtained from a local market.

### Obtaining canola phenolic extract

The canola pactol seeds were purchased from the Agricultural Research Center in Giza and then crushed in a coffee mill to obtain a finely divided material suitable for extraction studies. The canola meal (defatted) was subjected to different solvents with the aid of ultrasonic waves either by using an ultrasonic bath (USB), or by using an ultrasonic probe (USP). **First, by using an ultrasonic bath (USB)**: One gram of canola meal was added to 100 mL of different solvents 90% (acetone, ethanol, and methanol) in beakers in an ultrasonic bath for 30 min. Then centrifuge produces supernatant (1) The precipitate was redissolved with another amount of solvent then the first step was repeated to produce supernatant (2) Both supernatants were used for the chemical evaluations. **The second was by using an ultrasonic probe (USP)**: One gram of canola meal was added to the same solvents 90% (acetone, ethanol, and methanol) in beakers with the aid of an ultrasonic probe for 5 min, then centrifuge. The supernatant was used for the chemical evaluations. An appreciable amount of bioactive compounds for the encapsulation process was prepared by methanol 90% (proved to be the best among the tested solvents).

### Quantification and identification of bioactive compounds

#### Determination of soluble phenolic

The content of phenolic compounds was determined according to the method of^24^. 200 µL of the sample was completed to 3 mL distilled water. 2 mL of 10% Folin-ciocalteu reagent was added and then shaken well for 5 min. 1 mL of 7.5% sodium carbonate was added and then shaken. The mixture was left for 1 h in the dark then the absorbance at 765 nm was measured using a spectrophotometer (T80 UV vis spectrophotometers). The total phenol content of canola meal extracts is stated as mg gallic acid equivalent per gram dry matter (GAE/g).

#### Determination of total flavonoids

The colorimetric determination of soluble flavonoids was performed according to^25^. The absorbance at 510 nm was measured using a spectrophotometer (T80 UV vis spectrophotometers). Total flavonoid content of canola meal extracts expressed as mg quercetin equivalent per gram dry matter (QE/g).

#### Evaluation of antioxidant activity

Evaluation of the antioxidant activity by three methods (DPPH scavenging %, ABTS scavenging %, and FRAP mg/g).

##### DPPH radical-scavenging

The antioxidant activity of all soluble phases was evaluated by DPPH radical-scavenging. The method described by^26^ was utilized to determine the DPPH radical-scavenging. The reduction of the DPPH radical was measured at 517 nm. Results were expressed as percentage inhibition of the DPPH using the following equation:

$${\text{Inhibition of DPPH (\%) = (absorbance control}} - {\text{absorbance sample/absorbance control)}} \times {\text{100}}$$

where absorbance control is the absorbance of DPPH solution without extract.

##### ABTS radical cation scavenging assay

Free radical scavenging activity of plant samples was determined by ABTS radical cation decolorization assay according to^27^. ABTS^·+^ cation radical was produced by the reaction between 7 mM ABTS in water and 2.45 mM potassium persulfate (1:1), stored in the dark at room temperature for 12–16 h before use. ABTS^·+^ solution was then diluted with methanol to obtain an absorbance of 0.700 at 734 nm. After the addition of 5 µL of plant extract to 3.995 mL of diluted ABTS^·+^ solution, the absorbance at 734 nm was measured 30 min after the initial mixing.

The ABTS scavenging effect was measured according to^27^ using the following formula:

$${\text{Radical scavenging (\%) = [ab}}{{\text{s}}_{{\text{control}}}} - {\text{ab}}{{\text{s}}_{{\text{sample}}}}/{\text{ab}}{{\text{s}}_{{\text{control}}}}] \times 100$$

##### Ferric reducing antioxidant power (FRAP)

The reducing power of extracts was determined according to^28^. The absorbance was measured spectrophotometrically at 700 nm. The measurement was compared to the standard curve of a prepared BHT solution. The final results were expressed as milligrams of BHT equivalents per gram based on the dry matter.

#### HPLC analysis

HPLC conditions for analysis of extracted compounds using 90% methanol as the better-extracting solvent. HPLC analysis was carried out using an Agilent 1260 series. The separation was carried out using Eclipse C18 column (4.6 mm x 250 mm i.d., 5 μm). The mobile phase consisted of water (A) and 0.05% trifluoroacetic acid in acetonitrile (B) at a flow rate of 0.9 mL/min. The mobile phase was programmed consecutively in a linear gradient as follows: 0 min (82% A); 0–5 min (80% A); 5–8 min (60% A); 8–12 min (60% A); 12–15 min (82% A); 15–16 min (82% A) and 16–20 (82%A). The multi-wavelength detector was monitored at 280 nm. The injection volume was 5 µL for each of the sample solutions. The column temperature was maintained at 40 °C.

### Preparation of bioactive canola extract (BCE) gelling agents

Different solutions of BCE were prepared by dissolving 0.08, 0.2, and 0.4 g of BCE powder in 75 g of deionized water. Next, neat soy lecithin powder (15 g) was melted at 80 °C to form an oil phase. After that, the BEC solution was gradually added to the oil phase and mixed well. The mixture was homogenized by a high-speed homogenizer (T25, IKA, Germany) at 10,000 rpm for 1 min to ensure complete emulsification. The emulsion was pre-frozen at -20 °C for 24 h and then freeze-dried for 72 h to completely remove the water, which produced the BCE gelling agents powders with different BCE loadings (designated as BCE gelling agents). The final BCE concentrations in the gelling agents were 0.08%, 0.2%, and 0.4% w/w.

#### Encapsulation efficiency

The developed gelling powder’s encapsulation efficiency (EE) was determined through the method described by^29^ with some modifications. 0.45 g of developed gelling powder was dispersed in 5 mL of water and shaken for 10 min. Then, the non-encapsulated compounds (surface phenolic compounds) were centrifuged at 3000 rpm for 5 min. The total EE% was calculated according to the equations: -.

$$\begin{aligned} & {\text{Surface phenolic compounds (\%) = (Surface phenolic compounds/Total phenolic compounds)*100}} \\ & {\text{Total phenolic compounds EE (\%) = 100}} - {\text{Surface phenolic compounds}}\;{\text{(\%)}} \\ \end{aligned}$$

### Preparation and characterization of constructed oleogels using bi-oleogelator systems

**Fig. 1:**
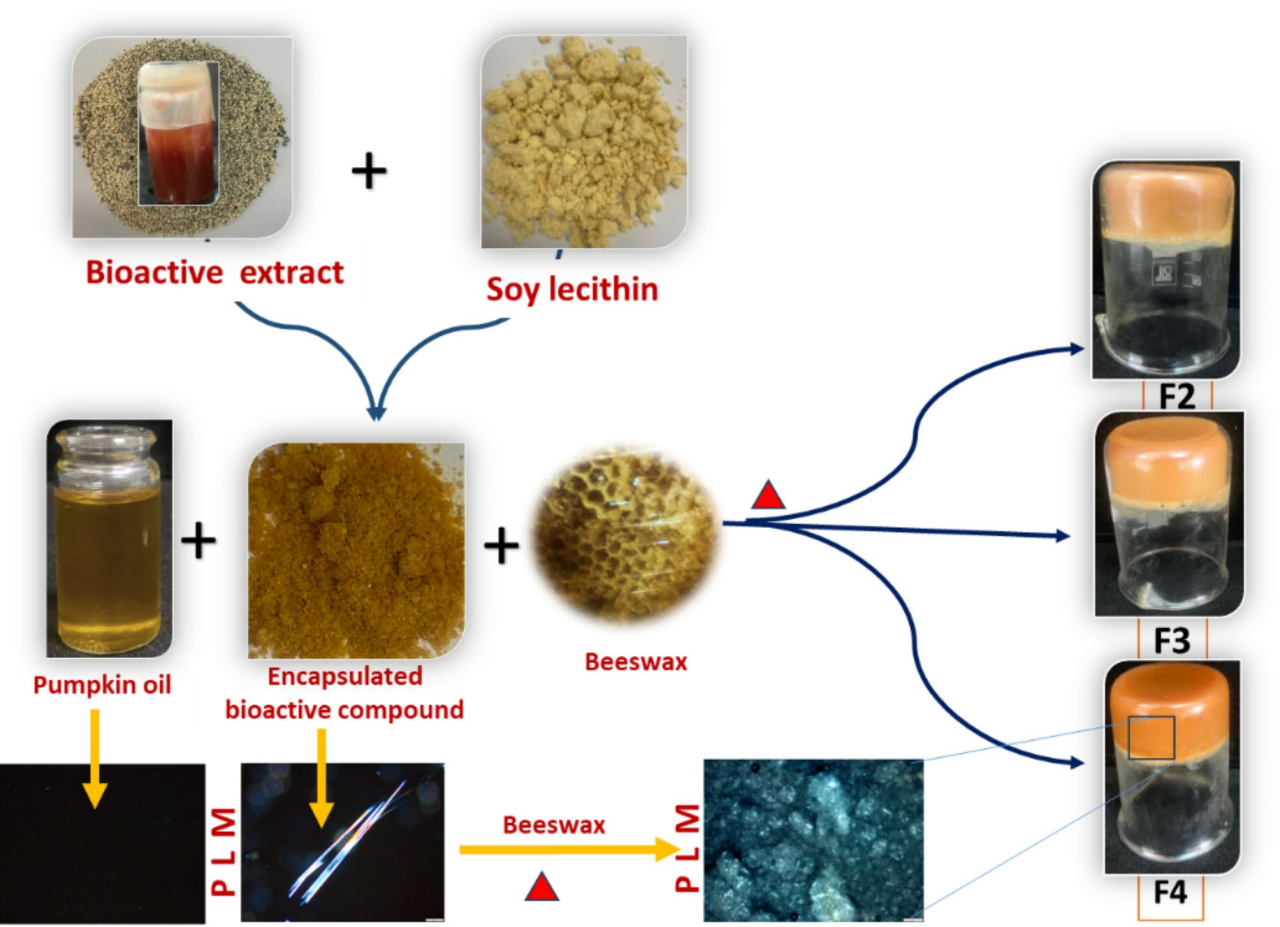
Workflow steps for preparation of constructed oleogels enriched with bioactive compounds.

Bi-oleogelator systems (Beeswax and BCE gelling agents) were used to develop constructed oleogels (Fig. 1). Beeswax at 5% (w/w) of oleogels was first completely dissolved in a water bath and added to pumpkin seed oil by stirring at 80 °C. BCE gelling agents at 15% (w/w) were gradually added to pumpkin seed oil containing beeswax at 80 °C using a magnetic stirrer to obtain a homogeneous mixture. BCE gelling agents were completely dissolved in the pumpkin seed oil during this process. After 20 min, the oleogel samples referred to as F2-F4 with different concentrations of BCE were placed in a refrigerator at 5 °C for 24 h. In addition, blank oleogel (defined as F1) with beeswax and soy lecithin without BCE was prepared as a control sample.

#### Polarized light microscope (PLM)

An automatic vertical microscope (Model BX51-P, Olympus, Japan) fitted with a professional video camera and a high-power LED lighting source was used to take polarized light micrographs of oleogel samples. Through a capillary tube, a drop of heated oleogels was put on the microscope slide, and the sample was then gently covered with the covers lip. Every sample was identified using a 20 × 10 magnification, and representative pictures taken in polarized light allowed us to see how the bi-oleogelator crystals were distributed throughout the oleogels.

#### Texture assessment

Texture profile analysis (TPA) was performed on samples using a double compression tester (Multitest 1d Memes in, Food Technology Corporation, Slinfold, W. Sussex, UK). All of the determined parameters (Hardness (N), Cohesiveness, Springiness (mm), Gumminess (N), and Chewiness (mJ) were determined using the International Dairy Federation’s (IDF, 1991) definition. The samples were prepared and measured as previously described by^30^.

#### Fourier transform infrared spectroscopy (FTIR) analysis

FTIR spectra of the functional groups of the developed oleogels were detected on FTIR Bruker Vertex 80v (National Research Centre, Egypt) with a resolution in the range of 4000–400 cm^− 1^.

#### Oxidation stability assessments

##### Peroxide (PV), p-anisidine (p-AV), and total oxidation value (TOV) measurements

The oxidation stability of oleogels was assessed using the Schaal oven test, with minor changes (Paradiso et al., 2010). The samples from every group were split equally into 100 mL sample bottles and kept in the oven for 60 days at a constant temperature (60 ± 2 °C). Every sample was kept sealed while being stored. During the Schaal oven test, the peroxide value and p-anisidine value were assessed every 10 days. PV was observed as a main oxidation product indication. The iodometric titration technique was utilized to measure the hydroperoxides following the official procedure of the AOCS (Cd 8b-90). As a sign of secondary oxidation products, p-AV was employed. The anisidine value was calculated using the official AOCS method (Cd 18–90). We performed each measurement in three duplicates. To systematically assess the oxidation characteristic of oleogel, the total oxidation value was estimated based on the variance of PV and p-AV, as shown in the following equation^31^:

1 $${\text{TOV}}=2{\text{PV}}+{\text{p}} - {\text{AV}}$$

##### Rancimat test of oleogels

A certain amount (3 g) of the oleogel samples was taken to investigate the thermal oxidative stability of developed oleogels using a Rancimat apparatus (Model 892 Professional Rancimat, Metrohm SA, Herisau, Switzerland) at 110 °C and 20 L/h airflow rate. The oleogels oxidative stability was determined through the time (h) equivalent to the induction period (IP).

##### Antioxidant capacity

The antioxidant activity of constructed oleogels was determined using DPPH; ABTS; and FRAP following the procedures mentioned in “Evaluation of antioxidant activity” section.

### Anticancer activity

#### Colorectal cancer cell lines

The colorectal cancerous HCT116 and Caco-2 cell lines were purchased from American Type Culture Collection (ATCC) supplied from VCSERA and cultivated in RPMI 1640 medium (Gibco) supplemented with 10% fetal bovine serum (Gibco), 100 U/mL streptomycin, and 100 U/mL penicillin at 37 °C in a humidified 5% CO_2_ atmosphere.

#### Cell viability assay

Colorectal cancer HCT116 and Caco-2 cells were seeded in 96-well plates at a density of 1 × 10^4^ cells/well and incubated for 24 h. Then, they were subsequently treated with 0, 25, 50, 75, and 100 µg/mL of pumpkin seed oil (R1), bioactive canola extract (R2), oleogels (F1-F4), and doxorubicin (DOX). Note that zero concentration represents the untreated cells. After that, the cells were incubated with 1 mg/mL of MTT reagent at 37 °C for 4 h and then it was discarded. The formed formazan crystals were dissolved using 100 mL of DMSO, followed by incubation and shaking. Finally, colorimetric analysis using a multiple reader was measured at 540 nm. The cell viability (%) was calculated and compared with control according to the previously reported protocol^32^.

#### Measurement of the half inhibitory concentration and fold change

The half maximal inhibitory concentrations (IC_50_) values, the concentrations inhibit 50% of cell viabilities, were obtained by plotting the percentages of cell viabilities versus the concentrations of the sample using polynomial concentration-response curve fitting models (OriginPro 8 software). On the other hand, fold change 1 (FC1) represents fold change of R1, R2, F1-F4 versus DOX (DOX/R1, R2, F1-F4) and fold change 2 (FC2) represents fold change of F1 versus R1 (R1/F1) and F2-F4 versus R2 (R2/F2-F4).

#### Time-dependent experiment

Colorectal cancer HCT116 and Caco-2 cells were seeded in 96-well plates at a density of 1 × 10^4^ cells/well and incubated for 1 h, 2 h, 3 h, 4 h, 5 h, and 6 h incubation times. Then, they were subsequently treated with 100 µg/mL of R2, F2-F4, and doxorubicin (DOX). After that, the cells were incubated with 1 mg/mL of MTT reagent at 37 °C for 4 h and then it was discarded. The formed formazan crystals were dissolved using 100 mL of DMSO, followed by incubation and shaking. Finally, colorimetric analysis using a multiple reader was measured at 540 nm. The time-dependent manner of the proposed treatment over colorectal cancer HCT116 and Caco-2 cells was measured.

#### Measurements of PI3k and COX-2 expressions

PI3k and COX-2 protein expression levels of HCT116 and Caco-2 cells up on treatments with the detected IC_50_ dosages of the proposed R2, F2-F4, and doxorubicin (DOX) were measured using ELISA kits purchased from (Wuhan Fine Biotech Co., China). These kits were based on a competitive ELISA detection procedure. The provided microtiter plate has been pre-coated with the target. During the reaction, the target in the sample or standard competes with a fixed amount of target on the solid phase supporter for sites on the Biotinylated Detection Antibody specific to the target. Excess conjugate and unbound samples or standard were washed from the plate, and HRP-Streptavidin-Biotin Complex (SABC) was added to each microplate well and incubated. Then 3,3′,5,5′-Tetramethylbenzidine (TMB) substrate solution is added to each well. The enzyme-substrate reaction is terminated and the color change is measured spectrophotometrically at a wavelength of 450 nm. The concentration of the target in the samples is then determined by comparing the OD of the samples to the standard curve.

#### Inducible nitric oxide synthase (iNOS)

*iNOS* enzyme activity of HCT116 and Caco-2 cells up on treatments with C1-C3 and F2, F4, and F6 were measured using an ELISA kit purchased from (Wuhan Fine Biotech Co., China). The competitive ELISA detection method served as the foundation for this kit. The target has already been pre-coated on the supplied microtiter plate. Target in the sample or standard competes for sites on the Biotinylated Detection Antibody specific to the target during the reaction with a set amount of target on the solid phase supporter. After cleaning the plate of excess conjugate and unbound sample or standard, each microplate well was filled with HRP-Streptavidin (SABC) and incubated. Each well then receives an addition of TMB substrate solution. After stopping the enzyme-substrate reaction, the color shift is detected using spectrophotometry at 450 nm in wavelength. The OD of the samples is then compared to the standard curve to ascertain the target concentration in the samples.

### Statistical analysis

Differences were determined with a one-way analysis of variance (ANOVA) followed by Duncan’s new multiple range test at *p* < 0.05 using Statistica 6 software (Stat Soft Inc., Tulsa, Oklahoma, USA), and the standard deviations (SD) were mentioned in corresponding figures.

## Results and discussion

### Quantification and identification of total phenolics, total flavonoids, antioxidant activities, and HPLC analysis

**Fig. 2:**
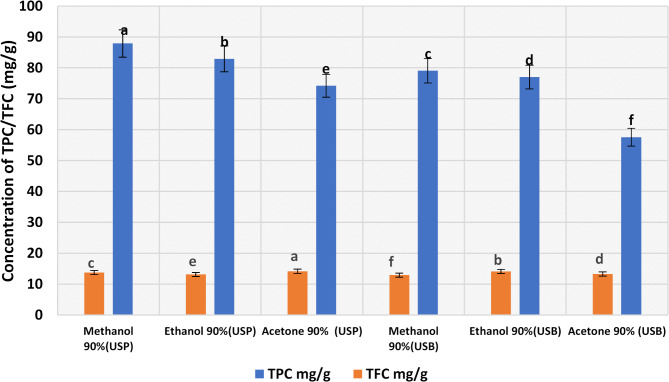
Effect of different solvents on the extraction yield of phenolic (mg GAE /g) and flavonoid (mg QE/g) compounds. Results are mean values of three replicates ± SD. A distinct superscript letter on the bars indicates a significant difference at *P* < 0.05.

There are various methods used in extraction procedures including maceration, decoction, infusion, digestion, percolation, superficial extraction, Soxhlet extraction, ultrasound-assisted, microwave-assisted, pressurized-assisted, enzymatic-assisted, and supercritical extraction^33,34^. This study investigated the effect of ultrasonic waves both by using an ultrasonic bath (USB) and by using an ultrasonic probe (USP) in the extraction of phenolic compounds which exhibited a significant effect on the extraction process. The bioactive contents of the canola meal extracted by (USB) and (USP) using 90% of each solvent (acetone, ethanol, and methanol) are provided in Fig. 2. It was observed that canola meal is rich in phenolics compared with flavonoids. Total phenolic compounds ranged from 57.53 to 87.88 mg/g and total flavonoid compounds ranged from 12.94 to 14.20 mg/g. The ultrasonic waves were effective in the extraction of bioactive compounds by the tested solvents, especially by using USP. Methanol 90% showed superior phenol extraction abilities (87.88 mg/g) especially while using an ultrasonic probe. Lower than that of the current study, the TPC of canola meal was 10–19 mg/g and the TFC was 1.47 to 1.95^35^. Higher than those in the current study, phenolic contents of canola and rapeseed husk crude extract varied from 128 to 296 mg/g^36^. Effective solvents for canola seed phenolics are aqueous methanol 70–100%^37^, or aqueous ethanol 65–75%^38^. The nature of the phenolic content extracted, pH, the type of extraction method, and the species of rapeseeds might all be factors in this variation in TPC values^39^.

**Fig. 3:**
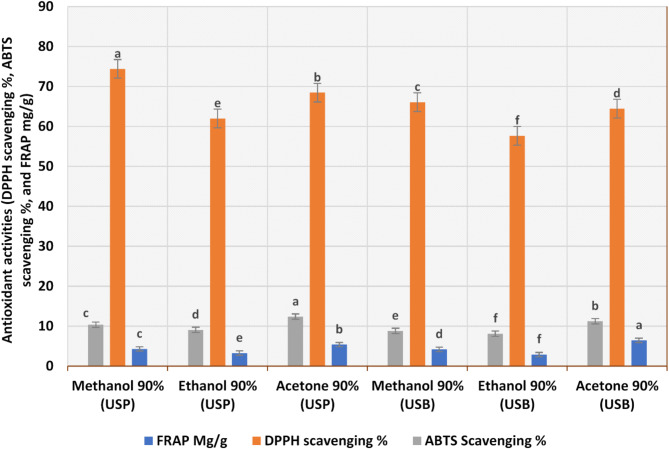
Antioxidant activities of different solvent extracts by three methods (DPPH scavenging %, ABTS scavenging %, and FRAP mg/g). Results are mean values of three replicates ± SD. A distinct superscript letter on the bars indicates a significant difference at *P* < 0.05.

Antioxidant activities of different solvent extracts by three methods (FRAP mg/g, DPPH scavenging %, and ABTS scavenging %) are provided in Fig. 3. All canola extracts showed antioxidant activities when measured by these methods. The methanolic extract of the canola meal was effectively scavenging the DPPH radical (74.42%) followed by acetone (68.4%) and then the ethanolicone (62%) utilizing the USP for the extraction process. Generally, all the methanolic extracts showed high radical scavenging activity. Almost all extracts demonstrate good ABTS radical scavenging activity especially acetone 90% showed the highest scavenging effect. The acetonic extract has the highest content of TPC and the most potent ABTS scavenging, anti-adipogenic, and pancreatic lipase inhibition^40^. Also, by using the FRAP method; almost all extracts exhibited reducing power ranging from 2.9 to 6.4 mg/g. Fadairo et al. (2022) showed how seed roasting by air frying can be a green pre-treatment method for valuing canola meal, producing the main derivatives of sinapic acid, and enhancing the extracts’ antioxidative capacity when measured by DPPH, FRAP, and by methanolic extracts^41^. Canola meal polyphenols as natural antioxidants can be used in the oil industry^35^.

The polyphenolic acid determined by HPLC is shown in Table 1. Canola meal methanolic extract contains chlorogenic acid, ellagic acid, gallic acid, methyl gallate, apigenin, and other polyphenols which were also observed by^42^.

**Table 1:** HPLC analysis of extracted compounds using 90% methanol as the better-extracting solvent.

Phenolic compounds	Conc. (µg/g)	Phenolic compounds	Conc. (µg/g)
Gallic acid	1265.49	Vanillin	737.20
Chlorogenic acid	1458.41	Ferulic acid	381.59
Catechin	52.90	Naringenin	29.73
Methyl gallate	18568.38	Resveratrol	544.79
Coumaric acid	46.62	Quercetin	252.08
Apigenin	1979.30	Cinnamic acid	13.62
Rutin	115.14	Kaempferol	113.90
Ellagic acid	1399.66	Hesperetin	48.26

### Encapsulation efficiency of gelling agents

The encapsulation efficacy of bioactive compounds encapsulated in soy lecithin with different proportions (0.08, 0.2, and 0.4%) was 98.1%, 96.4%, and 92.6%, respectively, using and freeze-drying. The encapsulating coating material considerably influenced the matrix’s capability to retain antioxidant phenolic compounds^43^. As noted in some earlier studies for the case of lecithin with antioxidants, the high values are most likely the result of hydrogen bonds forming between the active chemicals and the encapsulating materials^44^. Okumuş et al. (2021) investigated the use of soy lecithin as a substance for coating pomegranate peel ellagitannin extracts^14^. Rashidinejad et al. (2016) encapsulated epigallocatechin gallate and catechin in soy lecithin liposomes^45^. According to^46^, protein isolate and pullulan combinations had encapsulation efficiencies of 93.6% and 83.7% for quercetin and ferulic acid, respectively. The encapsulation efficiency was shown to be influenced by the types of phenolics and coated polymers.

### Characterization of constructed oleogels

#### Polarized light microscope (PLM)

**Fig. 4:**
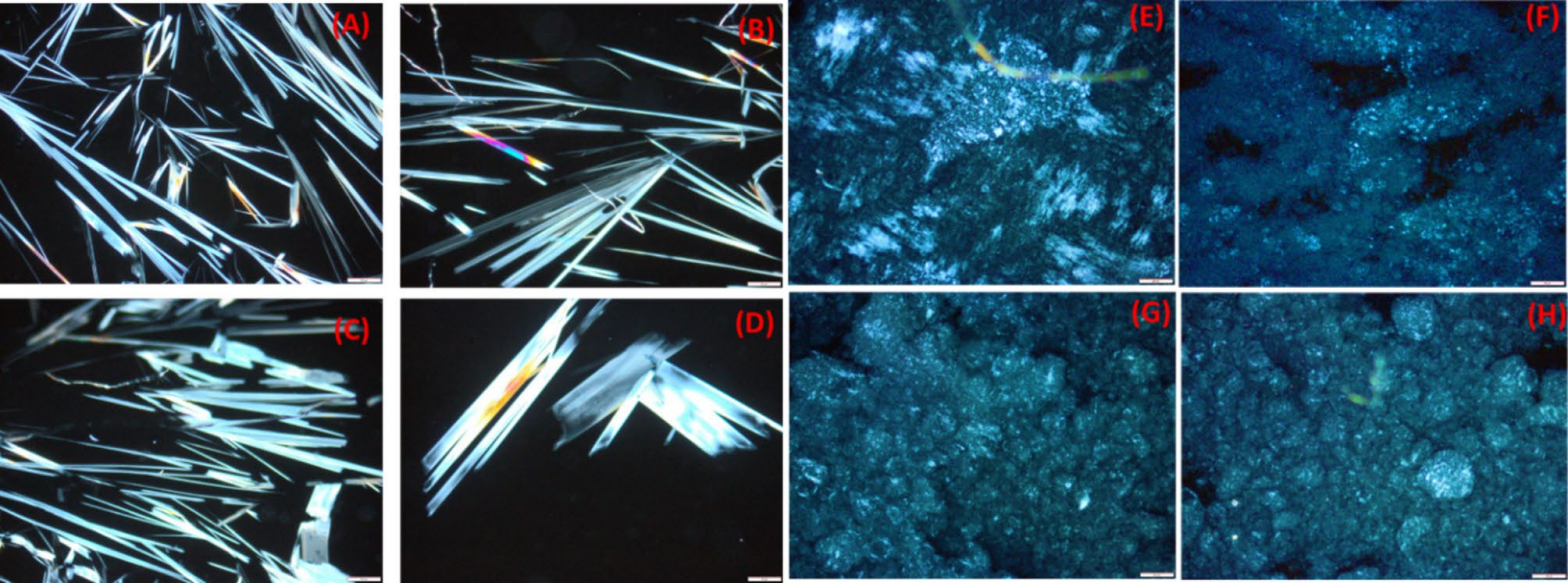
PLM images of (**A**) soy lecithin as plain, (**B**-**D**) bioactive extract encapsulated with different ratios in soy lecithin (BCE gelling agent) (**E**) plain oleogel and (**F**-**H**) developed oleogels using bi-oleogelator (15wt % BCE gelling agent 1 and 5wt%beeswax).

PLM images of encapsulated bioactive extract in soy lecithin (BCE gelling agent) and developed oleogels (F1-F4) using bi-oleogelators (15 wt % BCE gelling agent and 5wt%beeswax) are shown in Fig. 4. Soy lecithin with bioactive extract showed a fibrous structure with fibers formed by agglomerating soy lecithin worm-like inverted micelles into bundles that subsequently undergo branching and overlapping, forming the gel network (Fig. 4A-D). Using PLM, the microstructure of oleogels network was visualized. Oleogel samples formulated with beeswax and soy lecithin enriched with different ratios of the bioactive extract contained needle-like crystals (Fig. 4B-D). In agreement with the work of^47^ in oleogels, the crystals appeared as thin fibers that aggregated and displayed continuous branching. The crystalline network of bi-oleogelators gave oleogels a solid-like structure. As shown in Fig. 4E–H, the needle-like crystals composed of lecithin and beeswax were observed as birefringent spots in all of the samples. The crystal size of beeswax is tiny^48^. Combining lecithin with other oleogelators altered the packing geometry of the crystals by adsorbing on their formation sites, which in turn modified their crystal habit^49^. We concluded from Fig. 4 that the addition of bioactive extract did not affect crystal morphology because PLM pictures showed no noticeable change in crystal morphology when compared to normal oleogel. Oleogelators created the crystalline network of oleogel by published research^50^.

#### Texture analysis

The findings of the textural analysis of created oleogels are displayed in Table 2. By raising the bioactive extract concentration, the hardness of samples was decreased. In industry applications, hardness is a crucial property because it affects gel disintegration, which is closely linked to the release of bioactive extract. Comparing F4, its hardness was much lower than that of F1, F2, and F3. Concurrently, the hardness value of F1 was 112.50 g, which was somewhat more than F4. The findings demonstrated that the development of oleogels was significantly influenced by varying amounts of bioactive extract. Springiness, cohesiveness, gumminess, and chewing were also presented in Table 2. F4 had the highest values for previous items, while the highest value in gumminess was noted with F1. Cohesiveness and elasticity are associated with the gel structure’s ability to recover from deformation forces and how the structure is damaged following one. The internal structure of F1 was more readily degraded as a result, making them brittle but harder than F2-F3 which includes antioxidants. Elasticity and cohesiveness (elasticity) have an inverse relationship with food hardness^51,52^.

**Table 2:** Texture analysis of developed oleogels (F1-F4).

Samples	Hardness (*N*)	Springiness (mm)	Cohesiveness	Gumminess (*N*)	Chewiness (N*mm)
F1	112.50 ± 0.06	0.40 ± 0.09	0.09 ± 0.05	10.06 ± 0.03	4.05 ± 0.06
F2	56.40 ± 0.12	0.58 ± 0.05	0.12 ± 0.09	9.56 ± 0.12	3.79 ± 0.09
F3	27.35 ± 0.07	0.60 ± 0.12	0.29 ± 0.06	8.10 ± 0.09	5.15 ± 0.15
F4	19.50 ± 0.1	0.65 ± 0.06	0.45 ± 0.03	8.76 ± 0.12	5.66 ± 0.02

#### Fourier transform infrared spectroscopy (FTIR) analysis

**Fig. 5:**
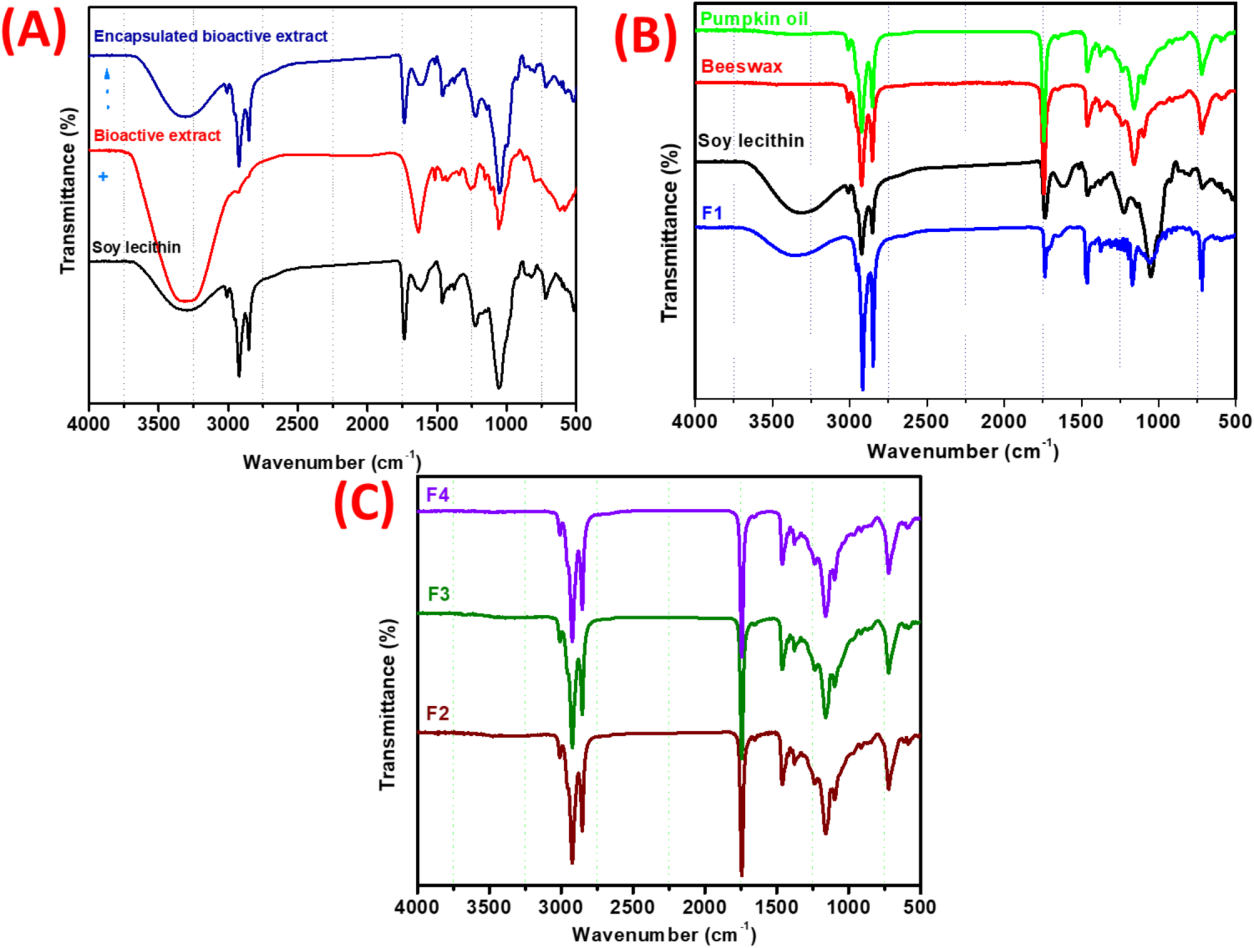
FTIR spectra of encapsulated bioactive compounds in soy lecithin and developed oleogels (F1-F4).

The chemical interactions between the ingredients in the oleogel formulation (pumpkin seed oil, soy lecithin, beeswax, and bioactive compound extract) were assessed using FTIR analysis. The functional groups of the components during the scanning range of 400 to 4000 cm^− 1^ exhibit important alterations, as depicted in Fig. 5. Changes in the shape, position, and intensity of peaks were indicators of these interactions^53^. Lecithin exhibited multiple different bands and peaks in its FTIR spectra. The wide band at 3432 cm^− 1^ is indicative of the stretching of OH. The symmetric and anti-symmetric stretching of the C − H bond (stretching vibration of the methylene group) is shown by the bands at 2922 and 2853 cm^− 1^. O stretching vibrations in the C double bond are responsible for the band at 1739 cm^− 1^. The methyl group’s C single bond H bending vibration is responsible for the peak located at around 1455 cm^− 1^. The P = O stretching vibration has a peak at 1228 cm^− 1^. Finally, the stretching vibration of P–O–C is represented by the peak at 1052 cm^− 1^^54^.

Additionally, The FTIR spectra of encapsulated bioactive extract (BCE gelling agent) were similar to soy lecithin, with new peak formation at 1516 cm^− 1^. A band at 1516 cm^− 1^ shows the aromatic C = C group and H-bonded stretching (prominent band) at the 3308 cm^− 1^ region is indicative of polyphenolic compounds^55^. Analysis of the FTIR spectra of F2-F4 confirmed interactions between encapsulated bioactive extract, beeswax, and pumpkin seed oil. According to^22^, gelators self-assemble to arrange the oil through physical interactions such as hydrogen bonding, p-p stacking, electrostatic interactions, and van der Waals forces. It was possible to distinguish several peaks, which correspond to the functional groups and vibration modes of the oil and bi-oleogelator. The peak at 3007 cm^− 1^ was related to the C–H stretching vibration of the cis-double bond (= CH) groups. The asymmetric and symmetrical stretching vibrations of the CH_2_ groups were found at 2924 cm^− 1^ and 2847 cm^− 1^, respectively. The spectra of the pumpkin seed oil and the oleogel samples (F1–F4) were comparable. The absence of the typical signal at 3312 cm^− 1^ in the oleogel samples indicates that hydrogen bonding is not present in those samples. The reason for this is that pumpkin seed oil is present in higher concentrations than beeswax and soy lecithin. Nonetheless, there may be some hydrophobic interactions between the hydrophobic portion of lecithin molecules and the non-polar oil molecules^56^. Additionally, the peak at 3007 cm^− 1^ indicated the presence of unsaturation in the produced oleogels. All of the oleogel samples had an absorption band that was comparable in the “fingerprint region,” which is between 1500 and 900 cm^− 1^.

#### Oxidative stability tests

##### Peroxide value, p-anisidine value, and total oxidation value measurements

***Peroxide value (first oxidation products determination)***

**Fig. 6:**
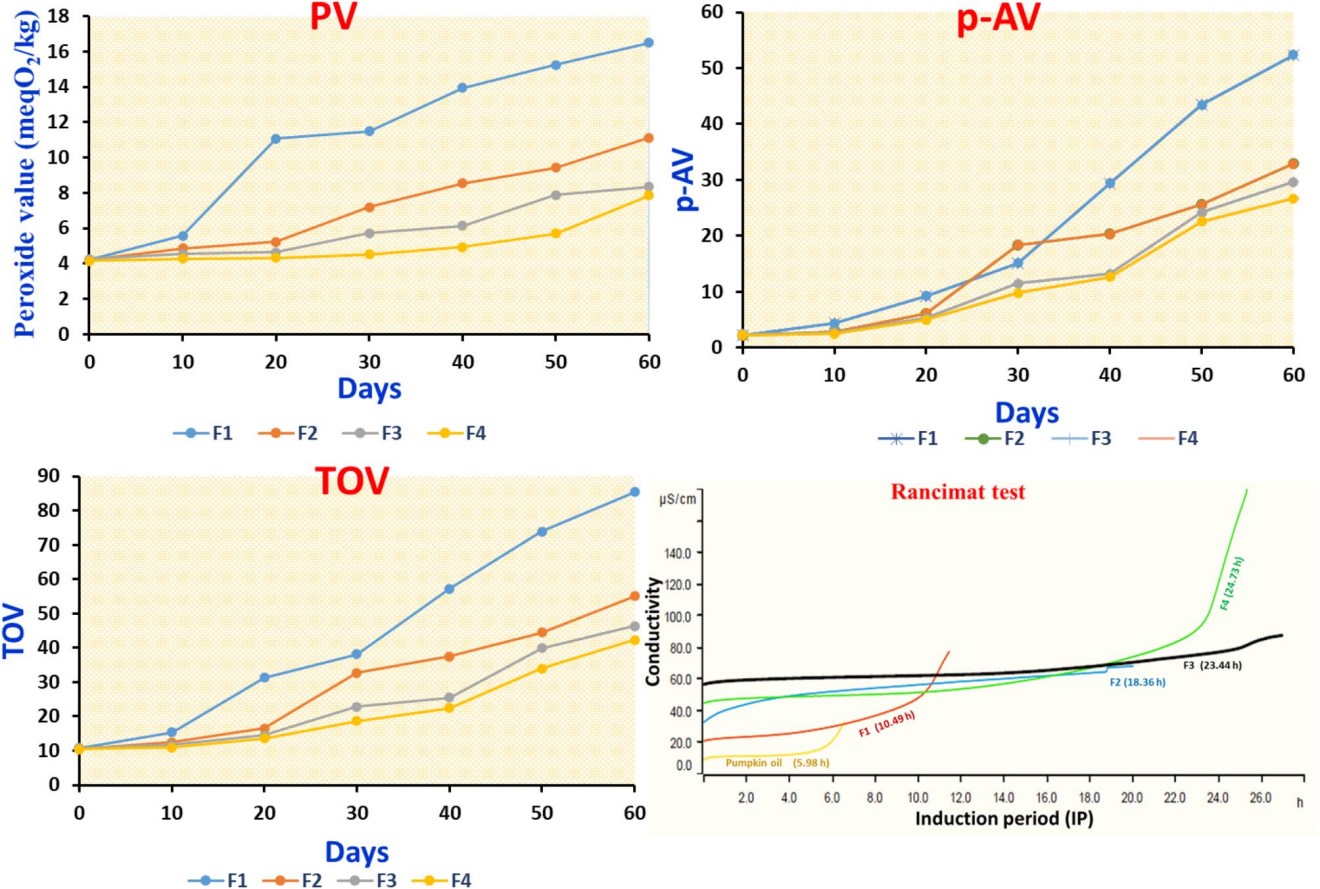
Oxidative stability tests including PV (primary oxidation indicator), p-AV (secondary oxidation indicator), TOV (2PV + p-AV), and Rancimat (thermal stability test).

The polyunsaturated fatty acids found in high levels in pumpkin seed oil are extremely prone to oxidative deterioration^17^. The first oxidation products that are produced throughout the oxidation process are evaluated by the peroxide value (PV), which also reflects the level of oxidation. It is a common indicator used to check the quality of oil and is derived from figuring out how much hydroperoxide is present during storage. The PV of the oleogels formed in the Schaal oven evaluation is displayed in Fig. 6. F1 was assessed as a control without the addition of bioactive compounds. It was clear that F4 had the best oxidative stability in primary oxidation over the 60-day storage period because its PV ranked lowest out of the three samples. In the 60 days, the PV of F1 was the highest, which was probably due to the lack of addition of bioactive compounds that have high antioxidant effects. The bioactive substances that were isolated from canola meal showed improved TPC in addition to raised antioxidant potential for scavenging free radicals, chelating metal ions, and converting ferric ions to ferrous ions—all of which are connected to lipid peroxidation (“Quantification and identification of total phenolics, total flavonoids, antioxidantactivities and HPLC analysis” section). Antioxidants are essential in the process of lipid peroxidation because they stop the peroxyl radical from propagating the radical chain by changing it into hydroperoxide^57^. Natural antioxidants can help stabilize lipid oxidation, as shown by^58^. Developed oleogels exhibited a similar trend of increasing PV with time of storage. But after day 50, there was a noticeable rise in the PV of F2. As expected at the studied temperature range, the bioactive extract was degraded as the preservation period increased. It appeared that oleogelation and bioactive extract worked in tandem to protect vegetable oil from oxidation. Days 10 to 60 saw a sharp rise in the PV of F1 to 16.51meq O_2_/kg, about twice as high as the PV of F4, suggesting that F1 has substantially oxidized. It is possible to suppose that the structure of the oleogels constructed by encapsulating bioactive extract with lecithin and beeswax restricted the mobility of the oil and protected it. Consequently, the resistance of F4 to oxidation was relatively steady while it was in storage. Oleogel formulations based on fish oil and curcumin were developed to preserve the lipid phase and stabilize curcumin as a bioactive substance^59^.

***P-anisidine value (secondary oxidation product determination)***

Hydroperoxides, as primary oxidation products, are converted to aldehydes and ketones (secondary oxidation products) during the oil oxidation process and are extensively utilized for the assessment of level oxidation^60^. One of the essential metrics for assessing the degree of secondary oxidation of fats and oils is P-anisidine value (p-AV). Figure 6 presents the change in p-AV throughout the 60 days. After 10 days, p-AV of F1-F4 increased to 4.30, 2.75, 2.69, and 2.43, respectively, indicating that F2-F4 was quite steady than F1 in the process of secondary oxidation. This demonstrated how a crystalline network formed by bioactive extracts encapsulated in lecithin and beeswax in oleogels might function as an “obstructor,” slowing down reactant flow and delaying the onset of chain propagation reactions. Our findings concurred with those of a previous investigation^61^.

***Total oxidation value***

The oxidative stability of oleogels was evaluated by estimating the total oxidation value (TOV) using PV and p-AV. The higher the oil quality, the lower the TOV value. The TOV of F1–F4 in Fig. 6 showed trends that were comparable to the PV. The TOV of every sample rose as the shelf life was extended. More specifically, compared to F3 and F4, the TOV of F1 and F2 increased significantly more rapidly. At the end of storage (60 days), the TOV of F2 and F3 reached 52.08 and 46.33, respectively. While the TOV of F4 was 42.35, it was almost lower than half of the TOV of F1 (85.36). The lowest TOV during storage was generally found in F4. One explanation for this result was that the antioxidant activity of the bioactive extract prevented hydroperoxide from forming. As this was going on, the crystal network prevented oil migration, which lowered secondary oxidation rates and ultimately lessened oleogel system oxidation^62^.

##### Rancimat

In addition, Rancimat tests were carried out as an additional technique for oxidative stability to demonstrate the outcomes of the primary and secondary oxidation products. By comparing the rate at which various oleogel induction periods (IP) decreased, the oxidation stability of oleogels was assessed^63^. The induction periods for F1-F4 samples were 10.49 h, 18.36 h, 23.44 h, and 24.73 h, respectively. The IP value of each oleogel across the entire storage process, as depicted in Fig. 6, revealed the order of F4 > F3 > F2 > F1, which was compatible with the findings of the oxidation product monitoring. F3 and F4 showed the highest oxidative stability based on this data. A considerable amount of antioxidant activity was demonstrated by the bioactive extract, with a particularly potent effect. This conclusion further confirmed the distinct effects of different concentrations of bioactive extract in the oleogel system and corresponded with the results of the PV and p-AV analyses. Experiments on oxidative stability showed that the oxidative stability of oleogels was greatly enhanced by the addition of bioactive extract. This finding suggested that the addition of different concentrations from bioactive extract oleogels could improve their shelf life.

##### Antioxidant activities (DPPH, ABTS, and FRAP) of constructed oleogels

**Fig. 7:**
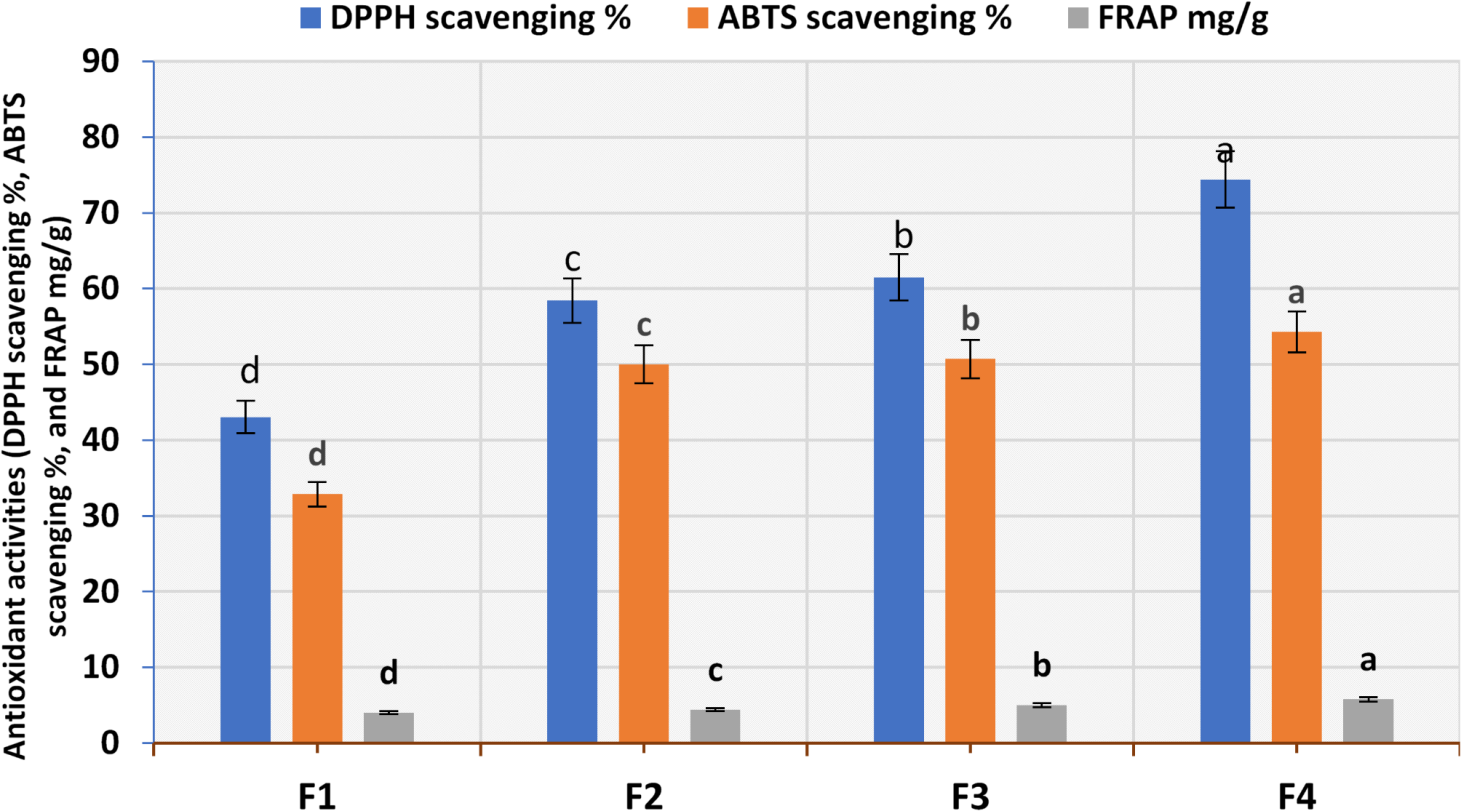
Antioxidant activities of control and prepared oleogel by three methods (FRAP mg/g, DPPH scavenging %, and ABTS scavenging %). Results are mean values of three replicates ± SD. A distinct superscript letter on the bars indicates a significant difference at *P* < 0.05.

Antioxidant activities of plain and oleogel enriched with different concentrations of bioactive extract were measured by DPPH, ABTS, and FRAP assays as shown in Fig. 7. Despite the different antioxidant capabilities measured by these assays, the total antioxidant capabilities of F4 were the highest, followed by F3, F2, and F1. The antioxidant activity values of F4 were74.40% for DPPH, 54.28% for ABTS, and 5.77 mg/g for FRAP, while the lowest values for plain oleogels (F1) were 43.05% for DPPH, 32.85% for ABTS, and 4.03 mg/g for FRAP. This phenomenon might be attributed to the addition of different concentrations of bioactive extract. The antioxidant activity of canola seeds is significantly influenced by phenolics, flavonoids, and α-tocopherols^64^.

### Assessment of cell viability of colorectal cancerous cells

**Fig. 8:**
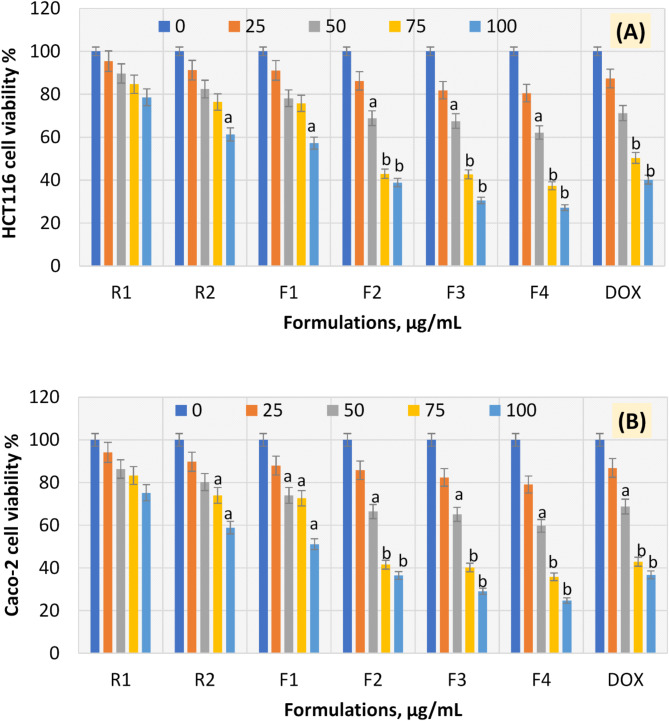
Colorectal cancer cell viability of HCT116 cell line (**A**) and Caco-2 cell line (**B**). Results are mean values of three replicates ± SD. A significant difference between the treated groups and the control at *P* < 0.05 is indicated by the letter “a” and at *P* < 0.01 is indicated by the letter “b”.

The cytotoxic effects of pumpkin seed oil (R1) and bioactive canola extract (R2) in addition to the four oleogel formulations F1-F4 were evaluated against colorectal cancer HCT116 and Caco-2 cells. The cytotoxicity in the current investigation was evaluated and exhibited in terms of cell viability percentage (Fig. 8) and IC_50_ values (Table 3). The results of these two evaluations accompanied by rationalization were interpreted. The results in Fig. 8 generally show that there is a manner of dose-dependent decrease in both cancerous HCT116 and Caco-2 cell viability percentage upon treatment with R1, R2, F1-F4, and DOX after incubation with 24 h (Fig. 8), noting greater cytotoxic effect on Caco-2 cell line compared to HCT116 cell line. That means that Caco-2 cells were more sensitive to the proposed therapeutic regimens than HCT116 cells. Intriguingly, F4 recorded the maximum cytotoxic effect against both cancerous cells, especially the Caco-2 cancerous cell line. The anticancer power of F4 may be due to the induction of reactive oxygen species (ROS) levels leading to the inhibition of cyclooxygenases-2 (COX-2) expression in cancer cells^65^.

From Table 3, the IC_50_ value of the R1 upon HCT116 cells was 90.03 µg/mL, while its formulation in F1 decreased the IC_50_ value to 81.73 µg/mL on the same cell line. When comparing R1 and F1 with DOX, the fold change was 0.78 and 0.85, respectively. The formulation F1 recorded a 1.10-time increase compared with R1. On the other hand, the IC_50_ value of the R1 upon Caco-2 cells was 87.76 µg/mL, while its formulation in F1 decreased the IC_50_ value to 78.08 µg/mL on the same cell line. When comparing R1 and F1 with DOX, the fold change was 0.75 and 0.85, respectively. The formulation F1 recorded1.12time increase compared with R1.

From Table 3, the IC_50_value of the R2 upon HCT116 cells was 83.71 µg/mL, while its formulations in F2, F3, and F4 decreased the IC_50_value to 65.87 µg/mL, 64.33 µg/mL, and 59.74 µg/mL on the same cell line, respectively. When comparing R2 and F2-F4 with DOX, the fold change was 1.06, 1.08, and 1.17, respectively. The formulations F2, F3, and F4 recorded1.27, 1.30, and 1.40-time increases, respectively, compared with R2. On the other hand, the IC_50_value of the R2 upon Caco-2 cells was 81.50 µg/mL, while its formulations in F2, F3, and F4 decreased the IC_50_ value to 64.23 µg/mL, 62.61 µg/mL, and 57.75 µg/mL on the same cell line, respectively. When comparing R2 and F2-F4 with DOX, the fold change was 1.03, 1.06, and 1.15, respectively. The formulations F2, F3, and F4 recorded1.27, 1.30, and 1.41time increases, respectively, compared with R2.

It was suggested that the cytotoxic impact of the proposed formulations may be due to the presence of phenolic compounds in the loaded bioactive canola extract (R2) in the three formulations (F2-F4). Some of these phenolic compounds exhibit potent antioxidant and anticancer properties. Interestingly, it is a rich source of sinapates and kaempferol analogues which are used as anticancer agents. Sinapates are transformed into vinyl phenol derivatives, such as canola, which have crucial uses in both food and the treatment of cancer. In addition to the bioactive canola extract (R2), pumpkin seed oil (R1) can support a healthy human diet^15^, because of its high concentration of antioxidant vitamins, phenolic components, carotenoids, tocopherols, phytosterols, and fatty acids. Intriguingly, it also has anticancer and immunomodulatory properties^16^. In parallel with our results, other phytochemicals such as epigallocatechin-3-Gallate, punicalin, punicalagin, and polyphenols recorded promising cytotoxic potential against various cancers through induction of apoptosis^66,67^.

**Table 3:** IC_50_ and fold changes of HCT116 and Caco-2 cell lines upon treatment with different formulations.

Treatment	HCT116 cells, IC_50_ (µg/mL)	FC1	FC2	Caco-2 cells, IC_50_ (µg/mL)	FC1	FC2
R1	90.03	0.78	Ref.	87.76	0.75	Ref.
R2	83.71	0.83	Ref.	81.50	0.81	Ref.
F1	81.73	0.85	1.10	78.08	0.85	1.12
F2	65.87	1.06	1.27	64.23	1.03	1.27
F3	64.33	1.08	1.30	62.61	1.06	1.30
F4	59.74	1.17	1.40	57.75	1.15	1.41
DOX	69.79	Ref.		66.18	Ref.	

FC1; means fold change of R1, R2, F1-F4 versus DOX (DOX/R1, R2, F1-F4).

FC2; means fold change of F1 versus R1 (R1/F1) and F2-F4 versus R2 (R2/F2-F4).

### Time-dependent experiment

**Fig. 9:**
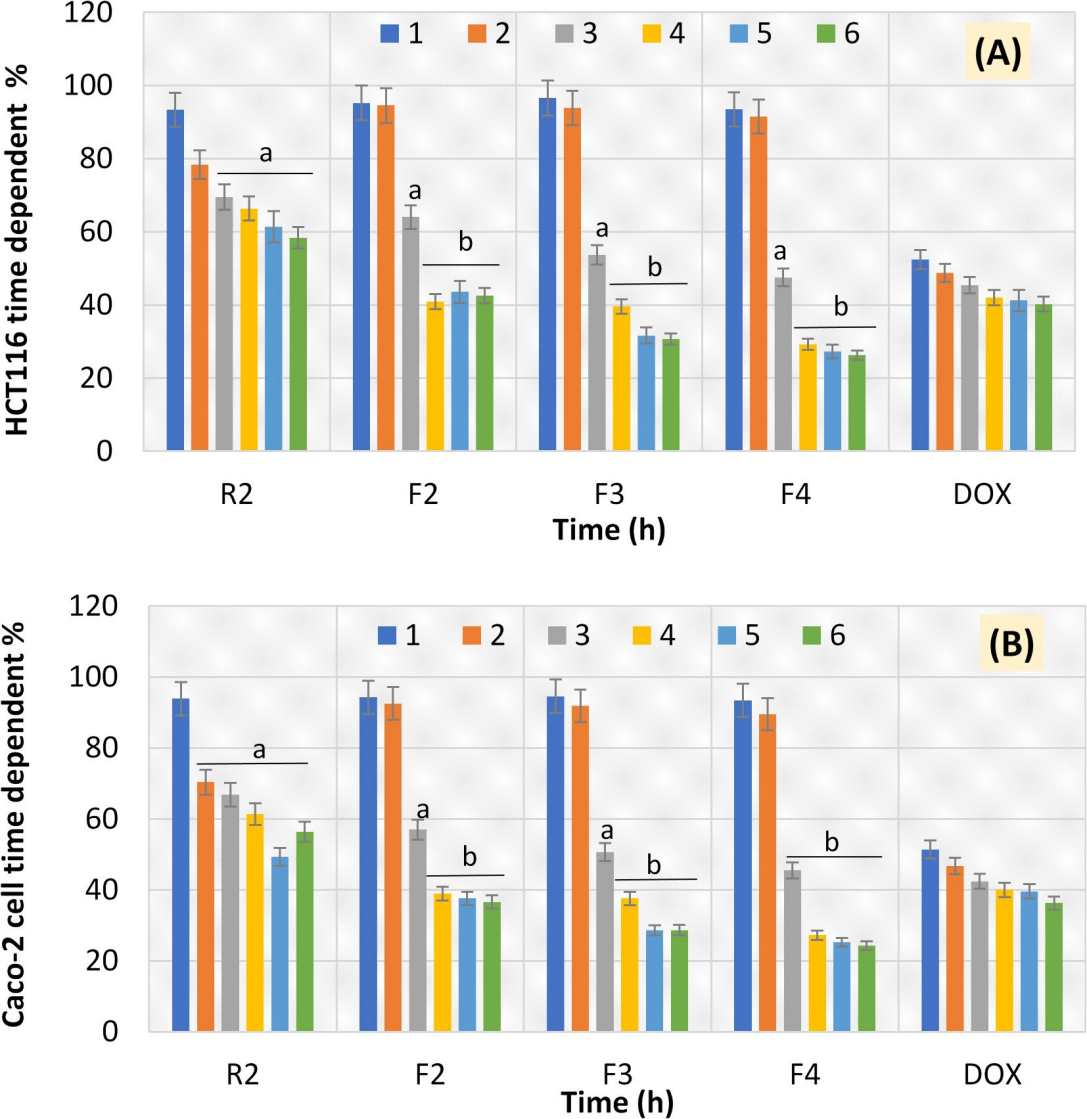
Bio-availability of F1-F4 and DOX against HCT116 cell line (**A**) and Caco-2 cell line (**B**) in a time-dependent manner (1–6 h). Results are mean values of three replicates ± SD. A significant difference between the treated groups (2 h to 6 h) and 1 h at *P* < 0.05 is indicated by letter “a” and at *P* < 0.01 is indicated by letter “b”.

To test the time-dependent manner of the proposed formulations F2-F4 versus R2 and DOX, we tracked both colorectal cancerous cells over 1–6 h. It was found that there was a gradual decrease over the 6 h incubation times in the time-dependent manner of HCT116 and Caco-2 cells upon R2 treatment until reaching 58.37% at 6 h. The same occurred in the case of DOX treatment until reaching 40.27% at 6 h. On the other hand, F2-F4 recorded no significant cytotoxic impact (*P* < 0.05) at 1 h and 2 h incubation times when applied on human colorectal HCT116 and Caco-2 cancerous cells, but started to be effective at 3 h incubation time. Intriguingly, the time-dependent manner of the F2-F4 therapeutic formulations reached the maximum beak of the cytotoxic impact on both cancerous cells at 4 h incubation time and continued constantly until reaching the 6 h incubation time. For HCT116 cells, F2 recorded 40.9% and 42.58%, F3 recorded 39.56% and 30.66%, and F4 recorded 29.22% and 26.27% at 4 h and 6 h, respectively. For Caco-2 cells, F2 recorded 38.91% and 36.59%, F3 recorded 37.57% and 28.67%, and F4 recorded 27.23% and 24.28% at 4 h and 6 h, respectively (Fig. 9). The promising time-dependent results of F4 may be due to the presence of beeswax-based oleogel with high percentage of bioactive canola extract (0.4%). Formulating R1 and R2 in the form of wax-based oleogels provides the proposed candidates with a plus advantage in tackling colorectal cancer cells. Because they can create a well-formed network with strong oil-binding properties even at low concentrations, wax-based oleogels are very effective^23^.

### Protein expressions of PI3k and COX-2

**Fig. 10:**
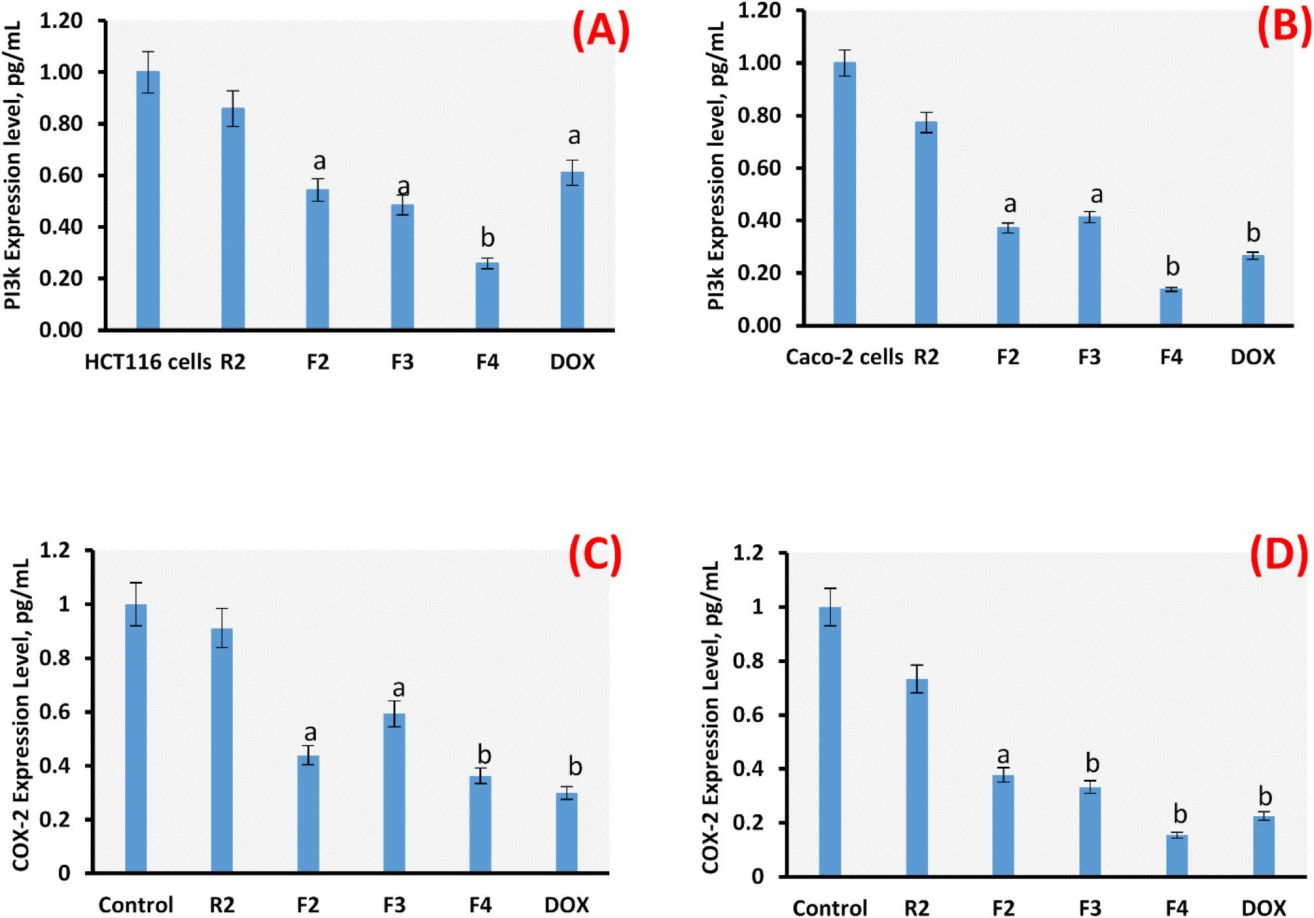
PI3k and COX-2 protein expression levels (pg/mL) using ELISA assays in HCT116 cell line (**A**, **C**) and Caco-2 cell line (**B**, **D**). Results are mean values of three replicates ± SD. A significant difference between the treated groups and the control at *P* < 0.05 is indicated by the letter “a” and at *P* < 0.01 is indicated by the letter “b”.

In some cases of colorectal cancer treatment, chemo-resistance develops leading to the reoccurrence of cancer, due to the induction of some survival (anti-apoptotic) proteins such as PI3k and COX-2. Therefore, the down regulation of these proteins is an attractive therapeutic plan, which is targeted in the present study. It was suggested that the introduced F4 may induce the greatest percentage of apoptosis through the down-regulation of PI3k and COX-2. It may be achieved by activating caspase-8, which upregulates Bax in the intrinsic pathway of apoptosis^68^. Figure 10 indicated that PI3k and COX-2 were down-regulated in colorectal cancer HCT116 and Caco-2 cells upon the applied treatments, recording the highest down-regulation for F4 compared to control in the case of Caco-2 cells. The relative expression levels of the PI3k and COX-2 proteins in both colorectal HCT116 and Caco-2 cells were down regulated in a different ratio upon R2, F2-F4, and DOX treatments compared to the control. R2 recorded 0.859 and 0.773 decrease levels in the PI3k protein expression in both colorectal HCT116 and Caco-2 cells, respectively, compared to the control (1). F2 recorded 0.543 and 0.371 decrease levels in the PI3k protein expression in both colorectal HCT116 and Caco-2 cells, respectively, compared to control (1). F3 recorded 0.485 and 0.412 decrease levels in the PI3k protein expression in both colorectal HCT116 and Caco-2 cells, respectively, compared to control (1). Intriguingly, F4 recorded 0.258 and 0.137 decrease levels in the PI3k protein expression in both colorectal HCT116 and Caco-2 cells, respectively, compared to the control (1). DOX recorded 0.0.611and0.266 decrease levels in the PI3k protein expression in both colorectal HCT116 and Caco-2 cells, respectively, compared to control (1). The down-regulation expression results of the COX-2 recorded the same pattern showed in PI3k, with the highest effect upon F4 treatment in both cancerous cells, especially in Caco-2 cells.

The anticancer power of F4 may be due to the inhibition of cyclooxygenases-2 (COX-2) protein expression levels and the elevation of reactive oxygen species (ROS) levels based on the following literature. Regarding colon cancer, the previous study was in agreement with our results indicating that dietary canola can decrease the tumor incidence and multiplicity. Mechanistically, inhibiting cyclooxygenases-2 (COX-2) protein expression in colorectal cancer is an important therapeutic target^65^. Thus, it was reported that the canola was able to significantly down-regulate COX-2 protein expression levels in colon cancer cells. Thus, it was concluded that dietary canola may be used as a chemopreventive agent for colorectal cancer through inhibition of COX-2 expression and elevation of ω-3 fatty acid levels^69^.

In parallel with our results, previous studies have examined the molecular mechanisms behind cancer cell death. It was reported that cell death may be accomplished through the induction of both intrinsic and extrinsic apoptotic lines in cancer cells by elevating the Bax/Bcl-2 ratio in the malignant cells^70^. On the other hand, cell death of the cancerous cells may undergo apoptosis via modifying noncoding RNA and changing epigenetics^71^. In general, it is important to track substances that alter the expression levels of anti-apoptotic proteins such as PI3k and Bcl-2.

### Evaluation of iNOS

**Fig. 11:**
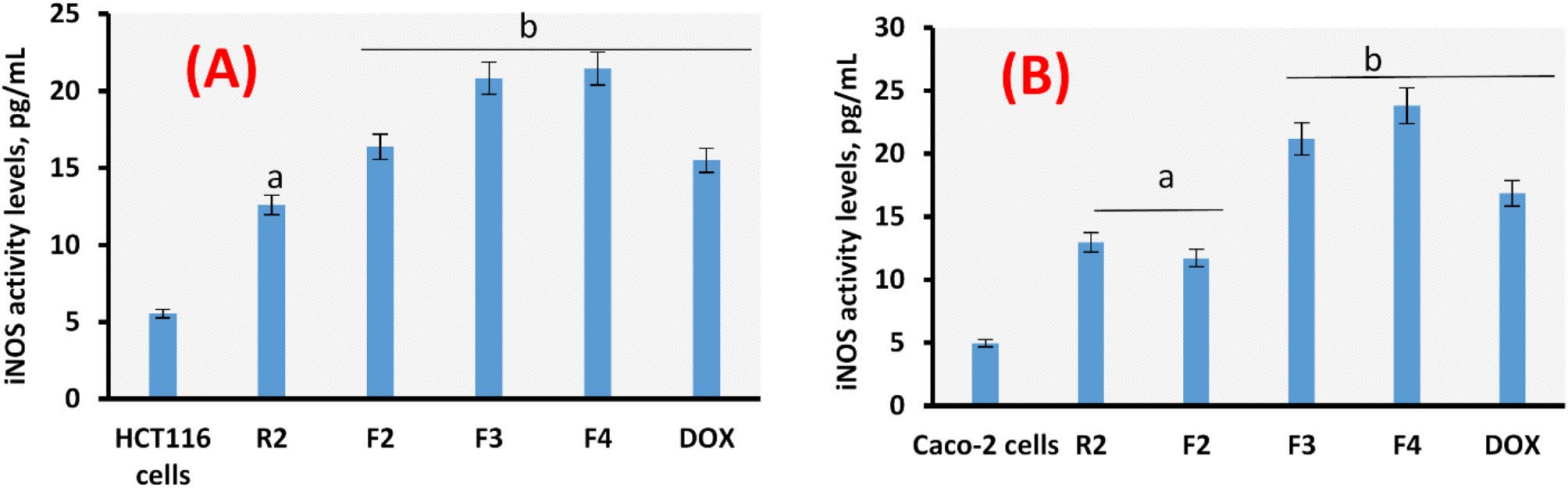
iNOS activity levels (pg/mL) using ELISA assay in HCT116 cell line (**A**) and Caco-2 cell line (**B**). Results are mean values of three replicates ± SD. A significant difference between the treated groups and the control at *P* < 0.05 is indicated by the letter “a” and at *P* < 0.01 is indicated by the letter “b”.

Figure 11a, b showed ROS inducer (iNOS) activity measurement in colorectal HCT116 and Caco-2 cells. It was noted that F3 and F4 induce high significant increase (*P* > 0.01) of iNOS enzyme activity levels in both cancer cell lines. High NO concentrations are produced by iNOS, which is linked to tumor inhibition. iNOS has been linked to apoptosis, cytotoxicity, and anti-tumor actions. The tumor-iNOS and other factors can have pro- or anti-tumor effects. There is a significant elevation of all treatments, this significant elevation was augmented (*P* > 0.01) in the formulation platform of F4. Intriguingly, Caco-2 cells were more sensitive to iNOS elevation than HCT116 cells. Another way of achieving high cytotoxicity against cancer, besides PI3k and COX-2 inhibition, may be based on ROS such as iNOS which was thought to be persuaded when the proposed phytochemicals were administered to the cancerous cells. Consequently, ROS-mediated cancer apoptosis is triggered. This tactic was accomplished by introducing the therapeutic chemical to cancer cells, where it interacted with DR5 receptors to sensitize the cells to a particular molecule^72^.

## Conclusion

This study demonstrated an efficient method to extract bioactive compounds from canola meal wastes with aqueous methanol (90%) using an ultrasonic probe. Bioactive canola extract gelling agents and beeswax were detected as good bi-olegelator systems to produce oleogels with high antioxidant activity. The oxidative stability of the oleogel systems was significantly enhanced due to oleogelation and bioactive extract working in tandem to protect oil from oxidation. There was no noticeable change in crystal morphology in developed oleogels. Oleogel with a high BCE concentration recorded the highest cytotoxic levels against colorectal Caco-2 cancerous cells through down-regulation of anti-apoptotic expression levels of PI3k and COX-2 and up-regulation of iNOS activity. Overall, the newly developed oleogels based on beeswax and BCE gelling agents are promising formulations. Thus, it can be applied for semisolid edible uses for delivering bioactive extract and as anti-colorectal cancer.

## Electronic supplementary material

Below is the link to the electronic supplementary material.

Supplementary Material 1

## Data Availability

Data is provided within the manuscript.
