# Supplementary material for: Anti-colorectal cancer activity of constructed oleogels based on encapsulated bioactive canola extract in lecithin for edible semisolid applications
Source: Sci Rep. 2025 Feb 10;15:4945. doi: 10.1038/s41598-025-88488-1 (PMC11811223; doi:10.1038/s41598-025-88488-1)
Supplement: Supplementary file 1 — Supplementary Material 1 [file 41598_2025_88488_MOESM1_ESM.docx]

**DSC thermogram of soy lecithin**

**The analysis of used soy lecithin according to Carl Roth Company is as follows:**

**- Appearance light brown to brown powder**

**- Phospholipids (Px31,5) ≥97.0 %**

**- Lysophosphatidylcholin ≤10 %**

**- Water ≤2.0 %**

**- Acid value ≤35**

**- Peroxide value ≤5.0**

**- Free fatty acid ≤0.5 %**

**- Made from genetically unmodified plants**

**Table: composition of different formulation of oleogels**

| **Formulations** | **Pumpkin seed oil % (w/w)** | **Bi-oleogelator** | |
| --- | --- | --- | --- |
|  |  | **Beeswax % (w/w)** | **BCE gelling agents% (w/w)** |
| **F1** | **80** | **5** | **15 (Soy lecithin only)** |
| **F2** | **80** | **5** | **15 (Soy lecithin with BCE 0.08)** |
| **F3** | **80** | **5** | **15 (Soy lecithin with BCE 0.2)** |
| **F4** | **80** | **5** | **15 (Soy lecithin with BCE 0.4)** |
